# 

**Published:** 1862-02

**Authors:** 


### DENTAL REGISTER

### OF THE WEST.

EDITED BY

J. TAFT AND GEO. WATT.

FEBRUARY–1862

**MONTHLY:**
**Three Dollars per Annum, in advance.**

CINCINNATI:
J. TAFT, PUBLISHER.

J. Taft, Dentist, No. 56 West Fourth Street.

